# A119 DEVELOPMENT AND EVALUATION OF LOW-COST GEL POLYPS FOR POLYPECTOMY SKILLS TRAINING IN NOVICE ENDOSCOPISTS

**DOI:** 10.1093/jcag/gwac036.119

**Published:** 2023-03-07

**Authors:** A Y Zhao, N Gimpaya, J Lisondra, R Fujiyoshi, Y Fujiyoshi, R Khan, D Tham, M A Scaffidi, R Bansal, C Walsh, S C Grover

**Affiliations:** 1 Division of Gastroenterology, St. Michael's Hospital; 2 Department of Medicine, University of Toronto; 3 Division of Gastroenterology, Hepatology, and Nutrition and the Research and Learning Institutes, The Hospital for Sick Children; 4 Department of Pediatrics, University of Toronto Faculty of medicine; 5 The Wilson Centre, University of Toronto, Toronto, Canada

## Abstract

**Background:**

Polypectomy is an essential skill for endoscopists to acquire. As polyps are encountered *ad hoc* during colonoscopies, exposure to polypectomy in clinical training may vary. There is a need to deliver a curriculum that standardizes exposure to polypectomy while remaining cost-effective for endoscopy programs worldwide.

**Purpose:**

To develop low-cost simulated polyps that can be incorporated into endoscopic training programs, and to evaluate their perceived realism and useability for polypectomy training.

**Method:**

We designed 3D molds based on the Paris classification, a validated rubric for polyp morphology. The polyps are depicted in Figure 1. Using low-cost materials, we created gel-based polyps compatible with physical colonic simulators. Current versions of the polyps were finalized based on visual realism and durability. Expert (performed >1000 procedures) and novice (<25 procedures) endoscopists were invited to perform simulated polypectomies and evaluate the realism of the polyps. Using a 7-point Likert scale (“strongly disagree” to “strongly agree”), we administered a survey adapted from the Direct Observed Polypectomy Skills (DOPyS) checklist to evaluate the polyps on practicality of design and useability for training. Additionally, the simulator’s resemblance to human polypectomy was assessed through a scale with 1 indicating “low resemblance” and 7 indicating “high resemblance”. The ease of identifying morphology was also evaluated, with 1 indicating “difficult” and 7 indicating “easy”.

**Result(s):**

The survey was completed by 11 expert endoscopists and 10 novices. The median score submitted by experts on the polyps’ useability in training the technique for mobilization of the polyp was 7 (IQR 6-7). Experts rated the simulator’s practicality in teaching cold snare or electrocautery techniques with a median score of 6 (IQR 6-7). Lastly, the ability of the simulator to develop skills in identifying and treating the residual polyp was assessed by expert endoscopists, giving it a median score of 6 (IQR 6-7). The simulators were tested on similarity to human polypectomy, with the median score of expert groups being 5 (IQR 5-6), and novice groups being 6 (IQR 6-6). Both groups were asked to rate if morphology could be identified using the simulator; the median score of expert groups being 6 (IQR 6-7), and 6.5 for novice endoscopists (IQR 5-7).

**Image:**

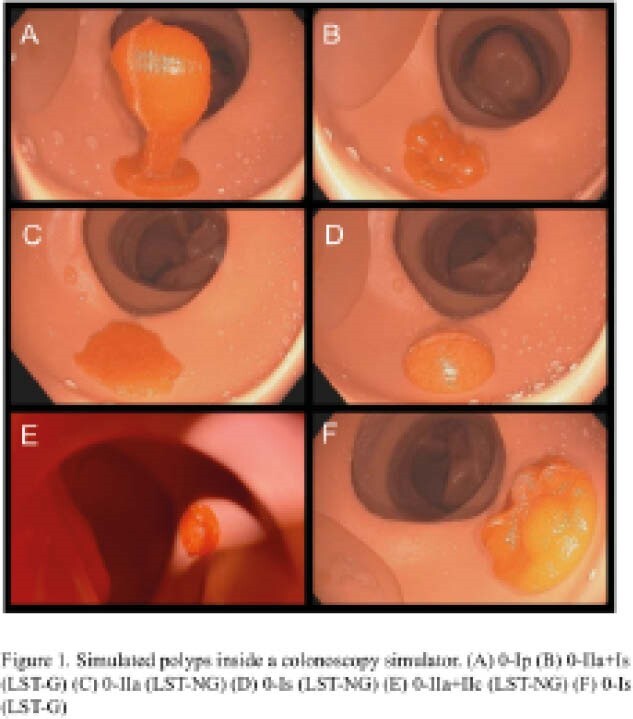

**Conclusion(s):**

The development of simulated polyps with differing morphologies using low-cost and common materials with high realism is feasible. These polyps may potentially be integrated into different endoscopic training programs.

**Please acknowledge all funding agencies by checking the applicable boxes below:**

None

**Disclosure of Interest:**

A. Zhao: None Declared, N. Gimpaya: None Declared, J. Lisondra: None Declared, R. Fujiyoshi: None Declared, Y. Fujiyoshi: None Declared, R. Khan Grant / Research support from: Rishad Khan has received research grants from AbbVie (2018) and Ferring Pharmaceuticals (2019) and research funding from Pendopharm (2019). , D. Tham: None Declared, M. Scaffidi: None Declared, R. Bansal: None Declared, C. Walsh: None Declared, S. Grover Shareholder of: Samir C. Grover has equity in Volo Healthcare., Grant / Research support from: Samir C. Grover has received research grants and personal fees from AbbVie and Ferring Pharmaceuticals, personal fees from Takeda, education grants from Janssen.

